# Atlantic SSTs control regime shifts in forest fire activity of Northern Scandinavia

**DOI:** 10.1038/srep22532

**Published:** 2016-03-04

**Authors:** Igor Drobyshev, Yves Bergeron, Anne de Vernal, Anders Moberg, Adam A. Ali, Mats Niklasson

**Affiliations:** 1 Southern Swedish Forest Research Centre, Swedish University of Agricultural Sciences, P.O. Box 49, 230 53 Alnarp Sweden; 2 Chaire industrielle CRSNG-UQAT-UQAM en aménagement forestier durable, Université du Québec en Abitibi-Témiscamingue (UQAT), 445 boul. de l′Université, Rouyn-Noranda, Québec, J9×5E4, Canada; 3 GEOTOP, Université du Québec à Montréal, CP 8888, Montréal, Québec, H3C3P8, Canada; 4 Department of Physical Geography, Stockholm University, Svante Arrhenius väg 8, 106 91 Stockholm, Sweden; 5 Centre for Bio-Archeology and Ecology (UMR5059CNRS), Université Montpellier 2, 163 rue Auguste Broussonet, F-34090 Montpellier, France

## Abstract

Understanding the drivers of the boreal forest fire activity is challenging due to the complexity of the interactions driving fire regimes. We analyzed drivers of forest fire activity in Northern Scandinavia (above 60 N) by combining modern and proxy data over the Holocene. The results suggest that the cold climate in northern Scandinavia was generally characterized by dry conditions favourable to periods of regionally increased fire activity. We propose that the cold conditions over the northern North Atlantic, associated with low SSTs, expansion of sea ice cover, and the southward shift in the position of the subpolar gyre, redirect southward the precipitation over Scandinavia, associated with the westerlies. This dynamics strengthens high pressure systems over Scandinavia and results in increased regional fire activity. Our study reveals a previously undocumented teleconnection between large scale climate and ocean dynamics over the North Atlantic and regional boreal forest fire activity in Northern Scandinavia. Consistency of the pattern observed annually through millennium scales suggests that a strong link between Atlantic SST and fire activity on multiple temporal scales over the entire Holocene is relevant for understanding future fire activity across the European boreal zone.

## Introduction

Fire is a primary driving factor of the ecosystem dynamics in the boreal forest, directly affecting global carbon balance and atmospheric concentrations of trace gases including carbon dioxide^1^,^2^. Large areas of the boreal forest that burn annually in North America (predominantly in Canada) and Eurasia (predominantly in Russia) exemplify a fundamental link between large scale atmospheric circulation processes and fire regimes^3^,^4^. Fires in the boreal region are estimated to represent around 9.1% of the annual total biomass burned globally^5^. Studies in the Eurasian boreal forest show that fire releases 4.8 to 15.4 Tg C/ha depending upon size and severity^6^. Climatically and human-induced changes in fire regimes impact the functioning of the boreal ecosystem by affecting regeneration and growth conditions for dominant tree species, forest composition, and its successional pathways^7^.

Under natural conditions, climate is the major factor controlling fire activity as large-scale circulation patterns determine periods of fire-conducive weather and synchronize fire regimes across landscapes and regions of the boreal zone^8^,^9^. Human activities have also impacted forest fire regimes^10^ through the use of fire for agricultural purposes such as slash-and-burn agriculture and, more recently, through fire suppression. Modern human impact results in a decrease in natural fire activity, even though increasing human densities are linked to a higher density of ignitions. Although in Scandinavia forest fires been historically one of the principal factors shaping the forests’ state and function^11^,^12^, forest fire activity in Northern Europe has been decreasing since the late 19th century^13^.

The European climate is largely determined by heat and humidity transfer from the adjacent North Atlantic Ocean, notably through the warm and saline North Atlantic Current (NAC) flowing northeastward^14^,^15^,^16^. The influence of the strong NAC results in relatively high winter temperatures and lower seasonal temperature contrasts as compared to the regions at similar latitudes in North America. Large scale circulation anomalies affecting the energy balance of the northern North Atlantic Ocean and associated atmospheric pressure patterns have been repeatedly shown to affect European weather^17^,^18^,^19^. Historically, however, most of the analyses have been focused on the winter climate, since it is the period when ocean-weather feedback appeared to be the most pronounced^20^. It is reasonable to hypothesize that the process affecting the humidity and energy transport over the North Atlantic Ocean should be equally important for the summer climate and, therefore, regional fire activity. Indeed, the summer temperatures and precipitation dynamics over Scandinavia have been shown to be largely controlled by the position of the westerly tracks and the strength of NAC^21^. Studies in different parts of the world have shown strong linkages between regional fire activities and changes in sea surface temperatures (SST) and the associated pressure patterns controlling precipitation over adjacent lands^22^,^23^,^24^,^25^,^26^,^27^.

In this paper, we hypothesized that ocean conditions in the northern North Atlantic play a determinant role on regional fire activity in northern Scandinavia^28^. We propose that a weakening of the NAC and stronger regional winter cooling, reflected by colder SST in the North Atlantic, would result in increased fire activity in the following fire season. We tested this hypothesis using modern forestry data on annually burned areas in northern Scandinavia (north of 60°N), multi-century and spatially-explicit dendrochronological reconstructions of annually burned areas, reconstructions of surface air temperature (SAT) and fire activity, and reconstructions of sea surface conditions (SST and sea ice cover) over the northeastern North Atlantic during the Holocene.

### The study region

The main study region covered the area of northern Sweden between 60°N and 70°N. It lies in the boreal climatic zone and receives air masses from the Atlantic region, especially during the winter season, which makes it particularly sensitive to the strength and thermal properties of NAC. Hence, despite the high latitudes, the mean January temperature ranges between −4 to −16 °C and the mean July temperature ranges from 12 to 16 °C. The number of days with a mean temperature above 5 °C ranges between 100 and 160^29^. The total annual precipitation is 600–700 mm on average, but with very high variability at higher elevations where it ranges between 400 and 1400 mm. Snow accounts for 30% to 50% of the annual precipitation and covers the ground for an average of 170–225 days/year^29^. The majority of modern forest fires in northern Sweden occur early in the fire season (May through June), although the total burned area is dominated by August fires^13^. Northern Sweden encloses the alpine zone, north, mid- and south boreal forests^30^. Scots pine (*Pinus sylvestris* L.), Norway spruce (*Picea abies* (L.) H. Karst), and birch (*Betula pubescens* Ehrh. and *B. pendula* Roth) are the dominant species in the forest vegetation cover. Fire has been the primary disturbance factor in this boreal ecosystem with stand-level fire return interval in mixed pine forests ranging between 30 to 100 years^12^. The modern (second half of the 20th century) fire cycle in the northern part of Sweden is 10^3^–10^4^ years^13^, which is attributed to efficient fire suppression policies.

## Methods

### Rationale and dataset selection

The starting point in the analyses was a pattern shown in a previous study, which demonstrated a relationship between major fire years in northern Scandinavia and regional positive temperature and negative precipitation anomalies^28^. We hypothesized that the fire pattern arises from large scale anomalies in atmospheric circulation, extending far outside the study region and beyond the fire season. The fact that the observed precipitation and temperature anomalies extended over most of the summer suggested that they were likely related to changes in energy states of the atmosphere and ocean surface. Accordingly, we extended the geographical frame of our analyses over the North Atlantic and included the winter and spring months preceding the start of the fire season. We tested the link between the surface ocean temperatures and fire activity at annual, centennial and millennial scales. Detailed analyses of spatial relationships were made using modern data from the 20^th^ century. We also extended the time interval for the analysis to the last 8000 years to analyse long-term changes but still avoiding climate perturbations related to the glacial-interglacial transition. Eight thousand years ago also roughly coincided with the start of paleo fire records in the study area (Supplementary Information Table SI 1).

In our long-term analyses, we largely capitalized on tree-ring and chironomid-based summer temperature reconstructions even though we realized that aridity records (e.g. precipitation) may provide equally important information about changes in historical burn rates. Reliable and sufficiently long paleo-aridity records are, however, not yet available for our study region. Seasonally-resolved precipitation records from tree rings are only available for the past few centuries^31^,^32^ and do not contain centennial signal^33^. Some precipitation records from lake sediment data are resolved with decadal resolution, but cover only a part of the Holocene^34^,^35^. By contrast, air temperature records from northern Sweden are available with seasonal (e.g.^36^) to decadal-centennial resolution for the entire Holocene^37^.

### Fire records with annual resolution

For the analyses of fire at annual scale, we used modern forestry statistics and dendrochronological fire reconstructions. Forestry statistics provided data on the annually burned forested areas in northern Sweden from 1942 to 1975 and from 1996 to 2014^13^. The composite fire record did not exhibit temporal autocorrelation (|r| < 0.10 for time lags up to 15 years).

**Figure 1:**
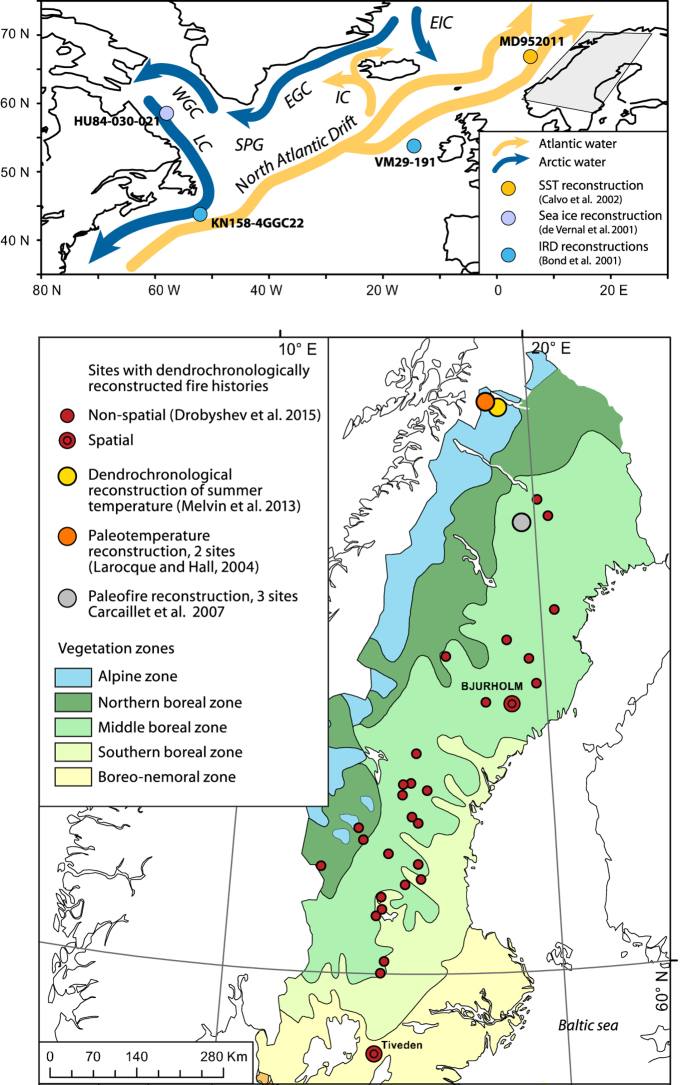
Map of the main current pathways in the northern North Atlantic and location of sites providing proxy data. LC Labrador current, WGC Western Greenland Current, EGC Eastern Greenland Current, IC Irminger Current, EIC Eastern Icelandic Current. The grey polygon of the upper panel refers to the map area of the lower panel.

Dendrochronological fire reconstructions, which aimed at extending annually-resolved fire records over centuries, were based on assigning the exact calendar year (crossdating) to fire scars in the wood of Scots pines (*Pinus sylvestris* L.). We used two dendrochronological datasets. First, we developed spatially explicit reconstructions of fire activity (amount of area burned annually) at two sites in northern Sweden, one since 1320 AD (site Bjurholm, area 60 800 ha, Fig. 1, Table SI 1) and the other from 1371 AD (site Tiveden, area 1200 ha). For both areas, we used the network of sites with independently reconstructed fire histories from tree fire scars. We considered properties of major fire breaks (e.g. lakes and rivers) and distribution of burned and unburned (and recording) sites across the landscapes to determine the size of each fire (for the detailed methodology, see^38^). Second, we used a previously published list of years which were shown to exhibit high fire activity at the regional scale (so-called *large fire years*, LFYs), identified by contingency analyses on a northern Swedish network of fire history sites (Fig. 1; Table SI 1 39). LFYs account for the majority of the forest burned area at decadal-centennial time scales^40^. LFY were identified using both the percentage of sites burned in a year and the theoretical probabilities of observing a particular number of areas burned in a year. We then assessed the theoretical probabilities though contingency analysis, considering fire activity as a realization of a binominal process:


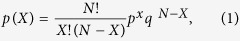


where N was the total number of recording areas in the analysis of a specific period; X – the number of burned areas in a single year; p – the probability of an area burning in any year, and q – the inverse of this probability. The differences between expected and observed frequencies were estimated with the Chi-square test (Sokal and Rolf 1995). We refer to^28^ for the details of this analysis.

### Fire records at above-annual resolution

As a proxy of forest fire activity in the past, we used charcoal concentration data from three ^14^C dated lake sediment cores from Northern Sweden (Fig. 1, Table SI 1 41). The cores were calibrated on age-depth models and were transformed to the same and constant time resolution (20 years^41^). We averaged the data from these sites through a standard two-step smoothing procedure using R package *paleofire*^42^ and converted the data into charcoal influx binned into 200 year intervals. Prior to averaging, the site chronologies were standardized to ensure that equal weights were attributed to each site in the master chronology.

### Surface air temperature (SAT) paleo reconstructions

As a source of annually resolved summer (May-August) temperature records over the last millennium, we used a reconstruction from the Torneträsk area in northern Sweden (Fig. 1), which included tree ring-width and maximum density measurements^36^. To extend back the temperature records over much of the Holocene, we used reconstructions of summer (May-September) temperatures based on the analysis of chironomid and diatom assemblages recovered from sediment cores of two lakes in Abisko Valley (Lake830 and Lake Vuoskku), northern Sweden^37^. Similar to the treatment of charcoal data, the initial chronologies were standardized and converted to the same temporal resolution (200 years) prior to averaging, to obtain a composite temperature record.

### Modern records and proxies of atmospheric and ocean variability

We used satellite-based sea-ice concentration data from the National Snow and Ice Data Center, Boulder Colorado US^43^, for the interval overlapping of the modern fire data (1996–2014). Sea-surface temperatures (SST) since 1870 were obtained from the UK Met Office Hadley Centre observations datasets^44^. We obtained monthly geopotential 500 hPa pressure heights (~200 km^2^ grid scale) from the NCEP/NCAR Reanalysis product from 1948 to 2014^45^,^46^. To represent fire-conducive weather conditions over the periods longer than those covered by fire statistics, we used a monthly 0.5° gridded dataset of self-calibrated PDSI (version 3.21^47^) over a northern Sweden area (60–64 °N and 4–14 °E), which are available for 1880–2010. In addition, we used monthly 0.5° gridded precipitation and temperature data (1901–2013) from the CRU TS3.22 dataset^48^.

As a paleo NAC strength indicator, we used an alkenone-based SST reconstruction from the Vøring Plateau, Norwegian Sea (core MD952011^49^). We also used two reconstructions of North Atlantic drift ice, available from coring sites in the northwestern (core KN158-4GGC22) and northeastern (core VM29-191) North Atlantic^50^. Percentages of Icelandic glass and percentages of hematite-strained grains were used as proxies for ice rafted debris (IRD) available from these sites. These chronologies were converted to the same temporal resolution (200 years), standardized and averaged to obtain a site-specific record of drift ice since 8000 cal years BP. We also used a dinocyst-based reconstruction of seasonal sea ice cover extent in the Labrador Sea (core HU84-030-021)^51^,^52^.

### Statistical analyses

We correlated (by using Pearson correlation) the chronology of 20^th^ century annually burned areas in Northern Sweden with each point in the SST and 500 hPa pressure fields for the area limited by 20–80° N and 80°W–30°E, and for the periods 1948–1975 and 1996–2014. Prior to the analyses, the fire data were log- and square-root transformed to ensure the normality of the distribution. Statistical significance of the correlations was tested through a two-sided Student t-test, with autocorrelation in the time series being taken into account through adjusting the critical values for the t-test^53^. For each correlation map, we also calculated field significance, a measure of correlation strength at the scale of the studied geographical area^54^.

Annual burned areas reconstructed from dendrochronological data were transformed to cumulative amounts over a moving average of 100-years with 10-year shifts, calculated over the period from 1350 (1400) to 1900 AD. This step resulted in smoothed fire chronologies at 10 year resolution to be used in regime shift analysis. The temporal limits of the analyses were defined by data availability (the start of the period) and the onset of fire suppression (the end of the period). The list of LFYs over the an area Northern Sweden, defined an LFY on the basis of contingency analyses and the synchronicity threshold over 1270–1914 AD^39^ was used in conjunction with the normalized tree ring-based reconstruction of summer temperature^36^. To this end, we computed the mean number of LFYs during “cold” and “warm” periods, as identified by regime shift analyses (see below), and then randomly shuffled the result with the replacement of the LFY occurrences 1000 times, thus obtaining a bootstrapped null distribution of random LFY occurrence during each of the warm/cold periods. We then constructed a 95% confidence interval around means and evaluated the statistical significance of the deviation of the observed empirical value as compared to the number expected under the assumption of random LFY occurrence.

To evaluate the spatial pattern of deviations in the Atlantic SST and pressure fields during 20^th^ century LFYs, we used superposed epoch analysis (SEA^55^). We selected the five LFYs known from the modern forestry statistics (years 1901, 1933, 1959, 1969, 2014)^13^. Although the replication for the SEA analyses was low, one should acknowledge an inevitable compromise between the notion of large fire year (i.e. how intense the fire activity should be to qualify a year as a *large fire year*, LFY) and the number of such years in analysis. Taking a more conservative approach towards the definition of LFY we therefore adopted a more opportunistic approach towards number of years, considered acceptable for the analysis. SEA analyses were done with the KNMI Climate Explorer tool^53^,^56^. SEA is based on aggregating data from years with a particular outcome (e.g. years with large forest area burned, LFY), averaging that data, and assessing significance of the departure of that average value from the mean calculated on the complete sampled distribution of the variable in question^57^. The method is designed to test for consistency in the response of a variable under or during a discrete event or factor, with significance being tested through bootstrapping or evaluating distribution of the complete sampled distribution.

To investigate the agreement of temporal changes in fire activity and those in reconstructed air and ocean temperature changes, we used a regime detection algorithm^58^. The algorithm focuses on the detection of time points of changes in the mean values of the respective time series and uses the Regime Shift Index (RSI)^59^, which is calculated for each point (*c*) of time series:


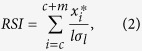


where *m* = 0, …, *l* – 1, with *l* as a cut-off length of evaluated regimes; σ_l_ is the average standard deviation for the complete set of one-year intervals in the original time series; and x_i_^*^ is a normalized deviation from the hypothetical mean level for a new regime.

To test for statistical significance in the RSI changes, the method relies on sequential *t*-tests, subsampling (Monte Carlo technique) and bias correction, which includes least squares estimates of the serial correlation to provide robust estimates of the significance of changes in the means^60^. A significance level of 0.1 and Huber’s weight parameter of 1, which controlled for weights assigned to outliers, were adopted for all of the analyses. This setting can be viewed as a threshold below which the sensitivity of the algorithm to detect potential changes progressively declines. Red-noise estimation was done through ordinary least squares estimation with data prewhitening with the length of sub-sample size being equal to 50% length of the defined cut-off.

We applied the algorithm independently for each of the analyzed time series: the northern Swedish fire activity, summer SAT reconstructions, eastern North Atlantic SSTs, IRD and Labrador sea ice cover chronologies. The analysis was performed for two time scales. For the dendrochronological reconstructions of annually burned areas (sites Bjurholm and Tiveden) and northern Scandinavian summer SAT, we used a 100 year cut-off length. For the SSTs, lake temperature estimates and charcoal-based fire reconstructions over the Holocene, the cut-off was set at 800 years. Prior to the analyses, SAT, SST, and IRD reconstructions were detrended by subtracting the long-term linear trend in order to suppress the influence from any trend over the entire analysis period.

## Results

### Relationship between modern fire activity, SST, and 500 hPa pressure heights

**Figure 2:**
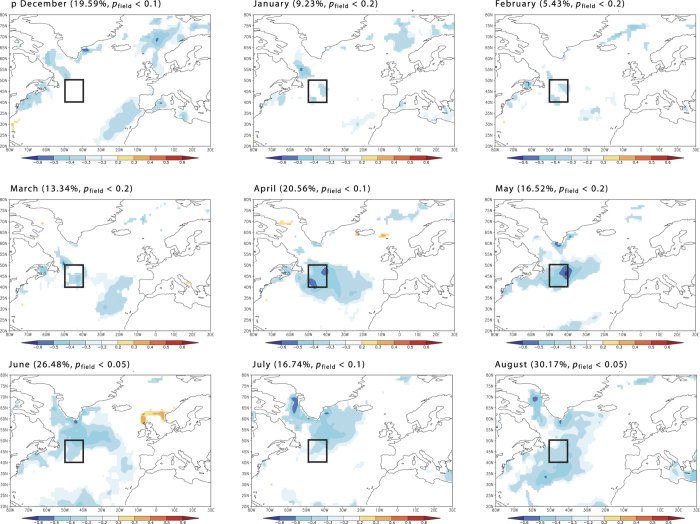
Correlation between annually burned forest area in Northern Sweden (north of 60° N) and monthly distribution of SSTs in the northern North Atlantic prior to and during the fire season. The area with the higher correlation (40–50°N, 50–40°W) is indicated by a square on all maps. Coefficients significant at *p* < 0.1 are marked by colour. Colour scale refers to the values of correlation coefficient. See Fig. SI 1C for the maps of significance levels.

Over the 20^th^ century, the annually burned area in northern Sweden correlated negatively with SST variations in parts of the extratropical Atlantic Ocean (Fig. 2). The region with the highest correlation (*r* in the range of −0.3 to −0.6) was observed within 50–40°W and 40–50°N, which is close to the Grand Banks, an area marked by a front between the Labrador Current and the NAC. The correlation was highest for April-May, which is the period of the year following the maximum sea ice spread in the northwest North Atlantic and immediately preceding the fire season. It persisted over the June through August, somewhat diminishing zonally by extending along the eastern coast of North America. The SST variability in April-May within the region off the Grand Banks accounted for nearly 40% of the variability in burned areas (Fig. SI 1A). Moreover, a moving correlation analysis indicated that the pattern was temporally stable (Fig. SI 1B). Although analyses of teleconnection between Northern Atlantic SSTs and fire activity showed moderate levels of significance at the scale of the selected geographical area (*p*_field_ < 0.05–0.20), this is a result of a limited geographical extent of the likely “centre of action” near the Grand Banks (an area surrounded by 40–50° N and 50–40° W) and the vast area included in these analyses (20–80° N and 80W–30°E). Consistently low p values in that “center of action” (p < 0.005, Fig. SI 1C) support the notion of teleconnection between North Scandinavian fire activity and SSTs.

Superposed epoch analysis based on the five LFY in the modern fire record (1959, 1966, 1969, 1997 and 2014) applied to the SST field pointed to the same important region of northern North Atlantic. However, the geographical and temporal extent of significant SST anomalies was more geographically limited than for the correlation analysis on the entire record, and it was temporally restricted to April-June (Fig. SI 2).

**Figure 3:**
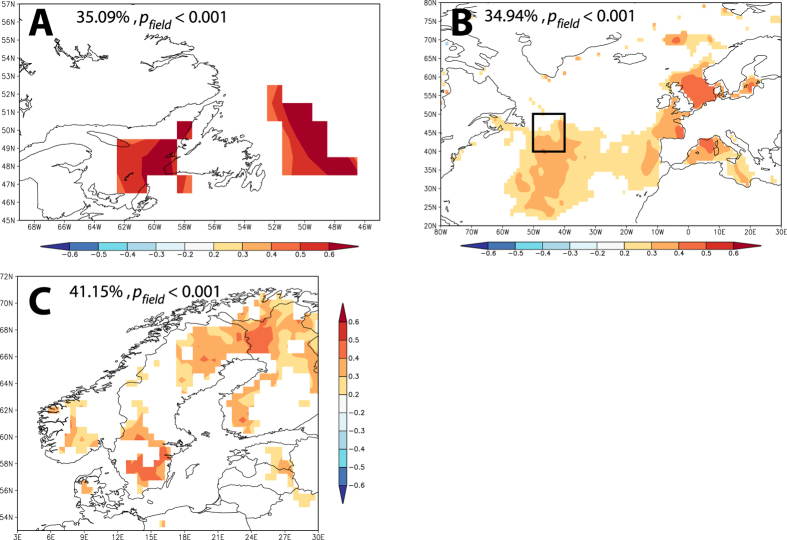
Teleconnection between the northern North Atlantic and fire weather in Scandinavia. (**A**) Correlation between annually burned area in northern Sweden and sea ice concentration (NSIDC dataset) around Newfoundland for February through April for the period 1996–2014. (**B**) Correlation between average April-May SST of the northern North Atlantic for the period 1870–2010 and self-calibrated PDSI for the area of northern Sweden (a region limited by 60–64 °N and 4–14 °E) for July,August. The area limited by 40–50°N and 50–40°W is indicated by a square. Note that lower PDSI values mean increased drought conditions. (**C**) Correlation between self-calibrated average July,August PDSI for the area of northern Fennoscandia and average April,May SST for area limited by 40 to 50°N and 50 to 40°W. In all graphs coefficients significant at *p* < 0.1 are marked by colour. Colour scale refers to the values of the correlation coefficients. The field significance and the fraction of the map with significant correlations (*p* < 0.05), are shown for each map. See Fig. SI 1D for the maps of significance levels.

There was a strong negative relationship between sea ice cover extent and SSTs in the northwest North Atlantic (r ~ 0.4–0.6, Fig. 3A), especially during the winter and spring. Therefore, we calculated the correlation between February-April sea ice concentrations in the Grand Banks areas and the burned areas: in the 48–52°N and 53–48°W region, a strong positive correlation linked the two variables (r ~ 0.5–0.7, Fig. 3A).

The negative relationship between SST and annually burned areas suggested that there might be a general relationship between SSTs in parts of the North Atlantic and the summer aridity of Northern Scandinavia. To test this hypothesis from modern instrumental data, we correlated the self-calibrated Palmer Drought Severity Index (PDSI) in July-August over Northern Sweden in the 60–64° N and 4–14° E region with Atlantic SST for April-May (Fig. 3B). The results suggested that a warmer extratropical North Atlantic was associated with higher PDSI, which corresponds to more humid and less fire-prone conditions in northern Sweden. Because the choice of the geographic region to analyze affects the results, we also ran the analysis using SSTs averaged over the area limited by 40 to 50°N and 50 to 40°W and correlated them with PDSI at each grid point over the whole Scandinavian Peninsula (Fig. 3C). The observed pattern showed a positive relationship between April-May SST and summer humidity for parts of the region, with the strongest teleconnections observed in southeastern Sweden, northern Sweden and northern Finland. The precipitation dominated this relationship (Fig. SI 3). During the 1900s, the annually burned areas in northern Sweden were correlated with precipitation rather than with temperature (Fig. SI 3A vs SI 3C).

**Figure 4:**
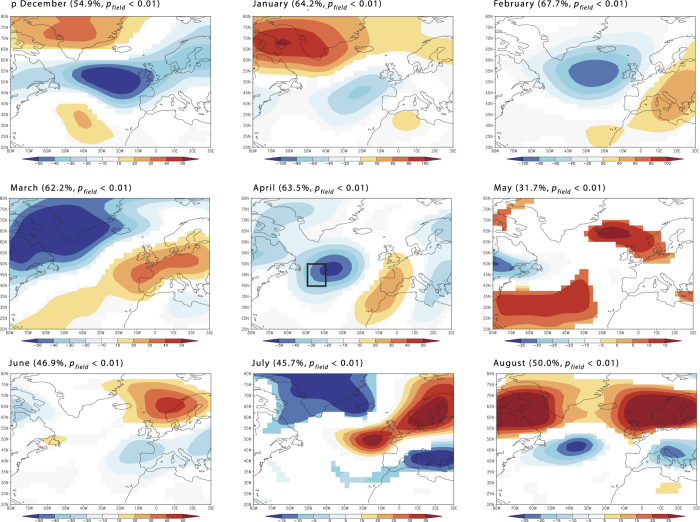
Superposed epoch analysis of monthly 500 mb pressure fields (NCER/NCAR dataset) for the five largest fire years in northern Sweden over period from December to August of the current fire season for 1948–1975 and 1996–2014. Areas with deviations significant at p < 0.10 are marked with colour . See Fig. SI 4C for the maps of significance levels.

During the fire season, high pressure systems dominated over Scandinavia and the British Isles, according to the SEA analysis (Fig. 4, Fig. SI 4). During the winter months, years with an anomalously large area burned were associated with negative anomalies in 500 hPa pressure heights, centred around 55° N across the Atlantic. Low pressure systems later developed over the Davis Strait, southern Greenland and northern Quebec during the spring (Fig. 4). Correlation analyses between 500 hPa pressure heights and annually burned areas, operating on the modern record, including both LFY and non-LFYs, revealed generally similar but a statistically less pronounced pattern (Fig. SI 4).

### Relationship between fire activity and temperature variability over 1400–1990 AD

**Figure 5:**
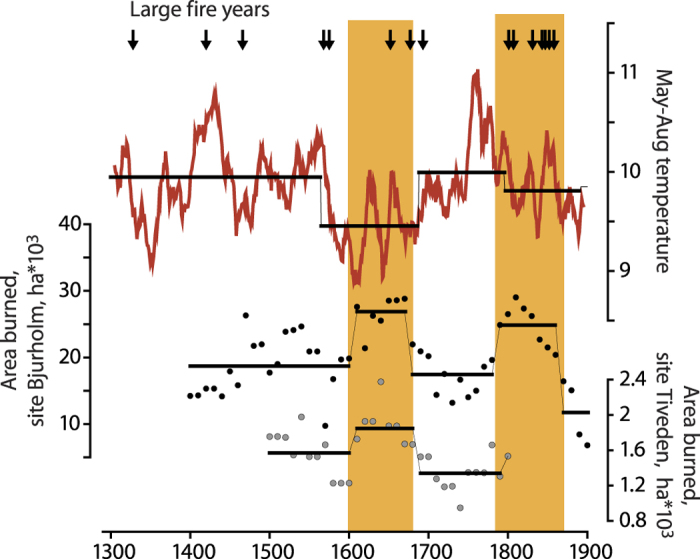
Dynamics of reconstructed fire activity and May–August temperature (running 11-year mean)^36^ in northern Scandinavia since 1300 AD. Thick horizontal lines represent periods of similar mean conditions identified through regime shift detection method based on sequential *t*-tests^58^ on original (unsmoothed) data points. Arrows indicate large fire years established through a contingency analysis on the network of sites with reconstructed fire histories (Fig. 1)^39^. Fire activity at sites Bjurholm and Tiveden is presented as cumulative annual area burned over a moving 100 year frame with a 10 year shift. Yellow bars indicate periods with increased fire activity.

Annually burned areas reconstructed at the Bjurholm and Tiveden sites revealed temporal synchronicity according to the regime shift analysis (Fig. 5), despite differences in the levels of average fire activity and site areas (Table SI 1). Fire prone intervals included the 1600s at both sites, and the 1800s for the Bjurholm site only. Lower average levels of fire activity were observed during the 1400–1600 period (1500–1600 in the shorter Tiveden record) and during the 1700s. Increased fire activity occurred during colder periods, as suggested by the regime shift detection algorithm applied independently to the tree-ring based temperature reconstruction (Fig. 5)^36^. Between1300 and 1880, LFYs occurred significantly more frequently during the cooler than warmer periods, according to the bootstrap analysis. The empirical difference between the number of LFYs in cold and warm periods (7) was larger than the 99.9% quantile (5) of the bootstrapped (n = 1000) distribution of that difference under the assumption of random LFY occurrence.

### Fire activity and environmental variability at Holocene scale

**Figure 6:**
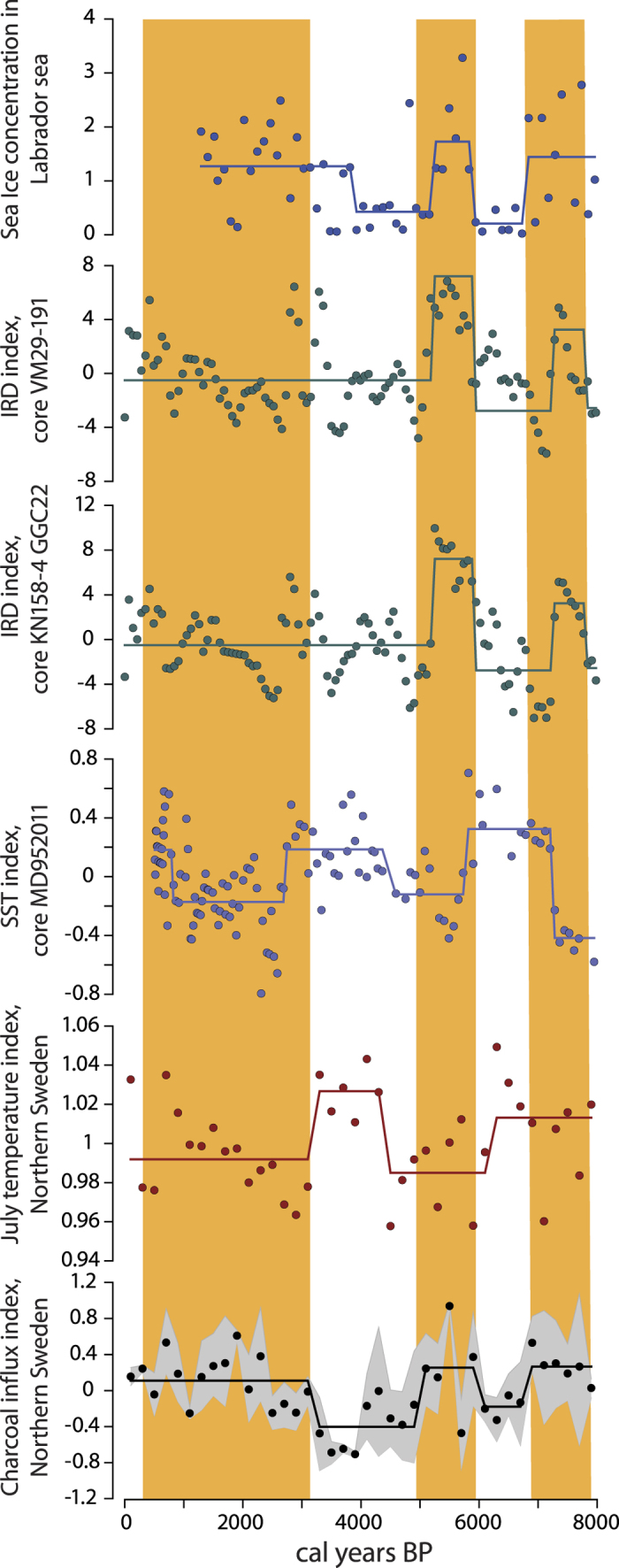
Holocene-long dynamics of fire activity and environmental proxies. Charcoal-based proxy of fire activity^41^ and chironomid-based temperature reconstruction^37^ represented conditions over northern Sweden. Sea-surface conditions are illustrated from SST reconstruction on the Vøring Plateau, Norwegian Sea core MD952011^49^, IRD occurrences in the northwest (core KN158-4GGC22) and northeast (core VM29-191) North Atlantic^50^, and sea ice cover estimates from the Labrador Sea core HU84-030-021^51^,^52^. See Table SI 1 for exact site locations. Charcoal reconstruction is an average of three lake sediment chronologies and chironomid reconstruction is an average of two reconstructions. Thick horizontal lines represent periods of similar mean conditions identified through regime shift detection method based on sequential *t*-tests^58^. 95% confidence envelop is shown for fire chronology. Yellow bars indicate periods with increased fire activity.

Northern Swedish fire activity since 8000 cal years BP as reconstructed from sediment charcoal^41^ tended to be inversely associated with chironomid-based July temperature estimates^37^ (Fig. 6), according to the regime shift analysis. Increased charcoal fluxes occurred between 8000 and 7000 cal years BP, 6000–5500 cal years BP, and since 3000 cal years BP. The two later intervals were associated with colder conditions, except for the earliest period when higher fire activity coincided with a warmer climate. However, increased fire activity broadly coincided with lower SST in the Norwegian Sea as inferred from alkenone-based reconstruction of core MD952011^49^ in all three intervals.

Sea ice cover reconstruction of the Labrador Sea from core HU84-030-021 revealed three periods of increased ice cover since 8000 BP, according to regime shift detection analysis (Fig. 6). These were 8000–7000 BP, 6000–5000 BP, and since 4000 BP until the present time. Increases in Labrador ice cover broadly coincided with increases in fire activity reconstructed from lake sediments in Northern Sweden. Although we observed a limited synchronicity between the Labrador ice chronology and the charcoal-based reconstruction of Scandinavian fire activity over 4000–3000 BP, we noted that during the latest period with increased fire activity (since 3000 calBP), the majority of data points representing ice reconstructions indicated above average ice concentrations.

Records of IRD, related to sea ice spreading in the northwestern (core KN158-4 GGC22) and eastern North Atlantic (core VM29-191), together with sea ice cover reconstruction from the Labrador sea (core HU84-030-021), indicated increases in winter sea ice concentration and its areal extent around 8000–7000 and 6000–5200 cal years BP. Both intervals coincided with increased fire activity in Northern Sweden (Fig. 6), although the regime shift analysis did not show the IRD index in the period since 3000 BP as significantly different from that between around 3000–5000 BP.

## Discussion

The correlation between the 20^th^ century fire activity in northern Scandinavia and SST and sea ice cover in the northwest North Atlantic pointed to a teleconnection between ocean conditions and the fire conductive weather of Northern Scandinavia. The oceanic region with the highest correlation is located off the Grand Banks, which is a critical area in the North Atlantic circulation due to the cold Labrador Current, flowing southward and making a front with the warm North Atlantic Current (NAC) flowing northeastward^61^,^62^. Hence, increased sea ice spread and freshwater export via the Labrador Current into the NAC^61^ may induce reduction of the Atlantic Meridional Overturning Circulation (AMOC). Superimposed epoch analyses on the northern Swedish LFYs during the 20^th^ century, suggested a close relationship of regional fire activity with winter sea ice cover and development of low pressure systems at the winter-spring transition (February-March) in the subpolar North Atlantic, further promoting southward sea ice transport from the Labrador Sea. A strong correlation between the annually burned area in Northern Sweden and the sea ice concentration off Newfoundland during the winter-spring months supported the hypothesis of a teleconnection between the oceanography of the northwest North Atlantic and hydroclimatological conditions in Scandinavia. Analyses of historical hydrographic data, reconstruction and modelling studies together suggest that sea ice and freshwater export through the Labrador Current is one of the important factors controlling the properties of the NAC at annual^61^, decadal^63^,^64^, centennial^65^,^66^, and millennial time scales^50^. The increased flow of cold and low saline Arctic water into the western North Atlantic weakens the NAC and leads to a southward shift of the western storm tracks^67^ fostering the development of high pressure systems over northern Europe (Fig. 4) with dominant dry Arctic air masses in summer. This is consistent with the negative correlation between summer aridity over most of the Scandinavian Peninsula and the average April-May SST in the northwest North Atlantic (Fig. 2A). This is also consistent with the results of modelling experiments simulating the response of the freshwater discharges on large-scale wind fields over the northern North Atlantic^65^. The critical role of North Atlantic SST and NAC on the aridity of Northern Europe has been further demonstrated from oxygen isotope data in late Holocene lacustrine record of Swedish Lapland: δ^18^O_si_ depletion events related to shifts in precipitation source from Atlantic δ^18^O_si_ - rich air masses to dryer Arctic air masses were concomitant with the IRD maxima in the northern North Atlantic^68^.

Changes in pressure fields over the North Atlantic apparently amplify the effect of SST on fire activity in northern Europe. Correlation analysis on SST fields suggested a strong relationship between average SST conditions and fire activity (Fig. 2) while showing a much weaker signal with respect to LFYs in SEA analyses (Fig. SI 2). The pattern was reversed in the analyses of 500 hPa pressure heights, which revealed a much stronger pattern during LFYs (SEA analysis, Fig. 4) than correlation analyses on the complete dataset (Fig. SI 4). We speculate that the relative contribution of SST dynamics vs. changing pressure patterns over the Northern Atlantic on fire activity in Northern Europe vary along a gradient in the regional fire hazard, with pressure anomalies being increasingly important during years of generally colder SST in the North Atlantic. Along with internal cycles and feedbacks within the subpolar gyre^66^,^69^, external forcings have been suggested to affect the flow of cold Arctic water to the northwest North Atlantic^50^,^70^,^71^. Based on detailed paleoceanographic reconstructions, linkages have been proposed between the decline in solar irradiance and the blocking of high pressure systems that modified storm tracks and weather over Western Europe^72^.

Annually burned areas dendrochronologically reconstructed for two areas in northern Sweden revealed strong synchronicity at centennial time scales. We observed increased fire activity during the 1600s and most of the 1800s, which were marked by surface air temperatures below average as independently reconstructed from tree-ring data (Fig. 5)^36^. This includes the coldest interval of the Little Ice Age (LIA) that was recorded in Scandinavia during 1600s. Cold intervals exhibited a higher frequency of LFYs as identified through synchronicity analysis of the North Sweden-wide network of fire history sites. We, therefore, propose that cool climate periods in Scandinavia were associated with low atmospheric humidity and increased frequency of high pressure systems dominated by cold and dry air masses. Such a hypothesis is supported by sunshine reconstruction in Fennoscandia indicating generally sunny summers during the LIA but cloudy conditions during the Medieval Warm Anomaly^73^. The negative correlation between surface air temperature and fire activity reported for the Northern Europe may not be unique. An increased fire frequency during the LIA has been reported from dendrochronological reconstruction in the boreal forest of Western Quebec and related to an increase in summer aridity^74^.

Colder periods in northern Scandinavia might be more prone to climatic extremes as compared to warmer periods, probably as a result of more unstable circulation modes and an increase in meridional flow^75^. We observed an association between cooler climate and the levels of fire activity while considered at the decadal through millennia scales (Figs 5 and 6). We speculate that the frequency of fire-prone periods likely increased under cool conditions due to both increased weather instability and the frequency of extreme weather episodes during the generally cooler periods. A study of precipitation variability in Sweden^33^ has indicated an increased variability at sub-decadal time scales during the LIA. Furthermore, low δ^18^ O_si_ values from lakes (suggesting an Arctic provenance of precipitation) have been noted for periods corresponding to low temperature as inferred from tree-ring records^35^,^76^. In addition, simulation experiments suggested increased intensity of storms during LIA^77^. On the annual scales, however, temperature during the fire season did not appear to correlate with annually burned areas in Northern Scandinavia (Fig. SI3).

Charcoal influx in lake sediments in Northern Sweden^41^, a proxy for regional fire activity, was synchronized with independently reconstructed regional temperature, SST dynamics of the Norwegian and Atlantic sea ice for an interval encompassing most of the Holocene (Fig. 6). Charcoal flux showed an inverse relationship with summer air temperatures reconstructed from diatom and chironomid assemblages in lake sediments^37^ and with SSTs of the Norwegian Sea. The data suggest higher regional fire activity during phases of NAC weakening and subsequent regional cooling. IRD from the northern North Atlantic^50^ indicated partial correspondence between paleo fire records^41^ and sea ice cover extent, which was negatively correlated to SST. In particular, an increase in charcoal flux coincided with an increased amount of sea ice cover in the Labrador Sea^52^ and IRD in the northern North Atlantic at 7000–8000 and 5000–6000 cal. years BP, whereas the 7000–6000 cal years BP interval corresponds to a decline in charcoal flux and minimum sea ice cover extent (Fig. 6). After 5000 cal years BP, the algorithm failed to detect periods with significantly different levels of IRD. The factors behind the loss of synchronicity between IRD and charcoal flux after 5000 cal. years BP remain unclear. However, we note that geographically closer estimates of climate variability such as August SAT reconstruction in northern Scandinavia and SST records from the Norwegian Sea, consistently exhibited a close correspondence with fire activity as inferred from lacustrine charcoal flux. Direct comparison between the lacustrine isotope record and the North Atlantic IRD also shows a good correspondence but mostly since about 2000 years^35^.

The proximity of cold and dry Arctic air masses makes the northern Scandinavian forests sensitive to climate variability. Because the southward expansion of Arctic air masses is largely controlled by the North Atlantic SSTs through the shift of westerly storm tracks, one should expect stronger teleconnections between ocean and fire activity in Northern Scandinavia than in more southerly located parts of the European sub-continent. Indeed, numerical experiments have revealed amplified differences in precipitation over Northern Europe from strong to weak AMOC^78^. This is consistent with the limited synchronicity of Holocene fire activity in northern^41^ vs. southern Scandinavia^79^, and with the different patterns of annual fire activity evidenced from both dendrochronological reconstructions^28^ and 20th century observational data^13^.

We propose that cold conditions over the northern North Atlantic, associated with low SSTs, redirect southward the precipitation associated with the westerlies and result in increased regional fire activity. Our study demonstrated a previously undocumented teleconnection between the dynamics of Atlantic Ocean and the regional fire activity in northern Europe. The consistency of results obtained from modern observations, dendrochronological, paleolimnological and paleoceanographical reconstructions suggests that teleconnection between North Atlantic SST and fire activity operated on multiple temporal scales during the Holocene, which is relevant for understanding future fire activity in Northern Europe. Two features of the regional climate likely divert fire activity from the a pattern of increasing with temperature. These are: (a) the dominating effect of precipitation on fire activity (Fig. SI 3C), and (b) the decoupling between SAT and precipitation (Fig. SI 3E) that results in fire activity mostly responding to precipitation and atmospheric humidity (Fig. SI 3D).

The link between SST and fire activity in Northern Scandinavia exemplifies a fundamental control of oceanic circulation on natural disturbance regimes in terrestrial ecosystems, as already demonstrated in other regions of the globe. Periods with large wildfires in boreal Canada and Alaska have been linked to the Pacific Decadal Oscillation (PDO), El Nino Southern Oscillation (ENSO)^26^,^80^,^81^ and global SST during the northern hemisphere winter^24^. In particular, the warmer phases of PDO have been shown to result in increased fire activity over the North American boreal forest. In Patagonia, years of increased fire hazard have been shown to relate to lower SST (La Niña events) leading to heavily reduced precipitation during winter and spring periods^82^. In Europe, no previous studies have examined the link between Atlantic SST and fire activity, although the predictability of European weather from ocean dynamics has been documented earlier^83^,^84^,^85^.

## Additional Information

**How to cite this article**: Drobyshev, I. *et al*. Atlantic SSTs control regime shifts in forest fire activity of Northern Scandinavia. *Sci. Rep.* **6**, 22532; doi: 10.1038/srep22532 (2016).

## Supplementary Material

Supplementary Information
