# Supplementary material for: Atlantic SSTs control regime shifts in forest fire activity of Northern Scandinavia
Source: Sci Rep. 2016 Mar 4;6:22532. doi: 10.1038/srep22532 (PMC4778016; doi:10.1038/srep22532)
Supplement: Supplementary Information [file srep22532-s1.doc]

Supplementary Information for

Atlantic SSTs control regime shifts in forest fire activity of Northern Scandinavia

Igor Drobyshev, Yves Bergeron, Anne de Vernal, Anders Moberg, Adam Ali, Mats Niklasson

Table SI 1. Metadata and references for the datasets used in the study. IRD - ice rafted debris.

| Dataset | Period covered | Reference /  Data source | Coordinates |
| --- | --- | --- | --- |
| Annually burned areas, modern times | 1942-1975 & 1996-2014 | ^1^ & ^2^ | Northern Sweden, ~ above 60 N |
| *Dendrochronological data* | |  |  |
| Large Fire Years | AD 1300 - AD 1900 | ^2^ & ref. inside | Northern Sweden, north of 60° N |
| site Bjurholm | AD 1320 - AD 1966 | ^3^ (see footnote A) | 63.93° N 18.81° E |
| site Tiveden | AD 1371 - AD 1884 | ^4^ (see footnote A) | 58.72° N 14.60° E |
| Summer temperature reconstruction | AD 815 - AD 2010 (see footnote B) | 5 | Northern Sweden |
| *Paleochronological records* | |  |  |
| Site Lövnäs | 9.5k y BP - present | 6 | 66.32º N 17.90º E |
| Site Raigejegge | 9.1k y BP - present | 6 | 66.16º N 18.21º E |
| Site Lattok | 10.1k y BP - present | 6 | 65.45º N 18.35º E |
| Lake830 | 9.5k cal y BP - present | 7 | 68.37º N 19.12º E |
| Lake Vuoskku | 10.5 k cal y BP - present | 7 | 68.33º N 19.10º E |
| *Ocean condition reconstructions* | |  |  |
| Ice cover, HU84-030-021 | 8.5k cal y BP - present | 8,9 | 58.37°N 57.51°W |
| IRD, VM28-14 | 11.5k cal y BP - present | 10 | 44.30º N 46.27º W |
| IRD, VM29-191 | 11.5k cal y BP - present | 10 | 54.27° N 16.78° W |
| SST, MD952011 | 11.8k cal y BP - present | 11 | 66.97° N 7.63° E |

Footnotes: A - spatial reconstructions of fire activity presented in the current study are previously unpublished; B - 10 samples and above.

Fig. SI 1. Regression between average April-May SST in the area limited by 40 to 50 °N and by 50 to 40 °W and annually burned area in the Northern Sweden (> 60 °N) for the 1942-1975 and 1997-2014 (A) and running correlations with bootstrap-generated significance envelops (B). Year 2014, the largest fire year in the modern history of Sweden, is circled. C - significance levels for the SST correlations, presented in the Fig. 2 of the main text. D - significance levels for the correlations presented in the Fig. 3 of the main text. Letters on the plates in Fig. D refer to the plates in Fig. 3 of the main text.

Fig. SI1 (continued)

1C.

Fig. SI1 (continued)

1D.

Fig. SI 2. Superimposed epoch analysis of the North Atlantic SST and annually burned area in northern Sweden for the periods 1942-1975, 1996-2014. Five largest fire years (total areas burned > 3700 ha) were selected as the event years. Areas with deviations significant at *p* < 0.1 are marked with colour.

Fig. SI 3. Relationships between annually burned area in northern Sweden (NS, the region above 60° N), average precipitation and temperature (CRU TS3.22 0.5° gridded dataset) and PDSI index ^12^ for July-August. A & B - correlation between the average July-August temperature and the area burned in NS (A) and PDSI (B); C & D - correlation between the average July-August precipitation and the area burned in NS (C) and PDSI (D); E - correlation between temperature and precipitation. For B, C, and E the correlations were computed in a point-wise fashion between two respective fields. F, G, H, I, J are maps with *p* values corresponding to the maps A through E, respectively. Colour scale refers to the values of the correlation coefficients (plates A through E) or p values (plates F through J) . The field significance and the fraction of the map with significant correlations (*p* < 0.05), is shown at the top of each map.

Fig. SI 3 (continued).

Fig. SI 4. Correlation analysis (A) and respective *p* levels (B) of the 500 mb pressure fields (NCER/NCAR dataset) over the northern North Atlantic and annually burned area in northern Sweden for the periods 1948-1975, 1996-2014. Five largest fire years (total areas burned > 3700 ha) were selected as the event years. C - Significance values for superposed epoch analysis (Fig. 4 of the main text) of monthly 500 mb pressure fields (NCER/NCAR dataset) for the five largest fire years in northern Sweden over period from December to August of the current fire season for 1948-1975 and 1996-2014. Areas with deviations significant at *p* < 0.1 (A) and at *p* < 0.05 (B, C) are marked with colour.

A.

Fig. SI 4 (continued).

B.

Fig. SI 4 (continued).

C.

References

1. MSB. Database on fires in Sweden. 2011.

2. Drobyshev, I., Bergeron, Y., Linderholm, H. W., Granstrom, A. & Niklasson, M. A 700-year record of large fire years in northern Scandinavia shows large variability and increased frequency during the 1800 s. *Journal of Quaternary Science* **30**, 211-221; *DOI:10.1002/jqs.2765* (2015)

3. Niklasson, M. & Granström, A. Numbers and sizes of fires: Long-term spatially explicit fire history in a Swedish boreal landscape. *Ecology* **81**, 1484-1499; *DOI:10.2307/177301* (2000)

4. Page, H. D. *et al.* Die Feuergeschichte des Nationalparkes Tiveden in Schweden. Eine kulturhistorische und dendrochronologische Untersuchung. *Forstarchiv* **68**, 43-50 (1997)

5. Melvin, T. M., Grudd, H. & Briffa, K. R. Potential bias in 'updating' tree-ring chronologies using regional curve standardisation: Re-processing 1500 years of Tornetrask density and ring-width data. *Holocene* **23**, 364-373; *DOI:10.1177/0959683612460791* (2013)

6. Carcaillet, C., Bergman, I., Delorme, S., Hornberg, G. & Zackrisson, O. Long-term fire frequency not linked to prehistoric occupations in northern Swedish boreal forest. *Ecology* **88**, 465-477; *DOI:10.1890/0012-9658(2007)88[465:LFFNLT]2.0.CO;2* (2007)

7. Larocque, I. & Hall, R. I. Holocene temperature estimates and chironomid community composition in the Abisko,Valley, northern Sweden. *Quaternary Science Reviews* **23**, 2453-2465; *DOI:10.1016/j.quascirev.2004.04.006* (2004)

8. de Vernal, A. *et al.* Dinoflagellate cyst assemblages as tracers of sea-surface conditions in the northern North Atlantic, Arctic and sub-Arctic seas: the new 'n=677' data base and its application for quantitative palaeoceanographic reconstruction. *Journal of Quaternary Science* **16**, 681-698; *DOI:10.1002/jqs.659* (2001)

9. de Vernal, A. *et al.* Dinocyst-based reconstructions of sea ice cover concentration during the Holocene in the Arctic Ocean, the northern North Atlantic Ocean and its adjacent seas. *Quaternary Science Reviews* **79**, 111-121; *DOI:10.1016/j.quascirev.2013.07.006* (2013)

10. Bond, G. *et al.* Persistent solar influence on north Atlantic climate during the Holocene. *Science* **294**, 2130-2136; *DOI:10.1126/science.1065680* (2001)

11. Calvo, E., Grimalt, J. O. & Jansen, E. High resolution U(37)(K) sea surface temperature reconstruction in the Norwegian Sea during the Holocene. *Quaternary Science Reviews* **21**, 1385-1394; *DOI:10.1016/S0277-3791(01)00096-8* (2002)

12. Dai, A. G. Characteristics and trends in various forms of the Palmer Drought Severity Index during 1900-2008. *Journal of Geophysical Research-Atmospheres* **116**; DOI:10.1029/2010JD015541 (2011)
